# Cell-Free DNA and CXCL10 Derived from Bronchoalveolar Lavage Predict Lung Transplant Survival

**DOI:** 10.3390/jcm8020241

**Published:** 2019-02-13

**Authors:** Joshua Y.C. Yang, Stijn E. Verleden, Arya Zarinsefat, Bart M. Vanaudenaerde, Robin Vos, Geert M. Verleden, Reuben D. Sarwal, Tara K. Sigdel, Juliane M. Liberto, Izabella Damm, Drew Watson, Minnie M. Sarwal

**Affiliations:** 1 Department of Surgery, University of California San Francisco, San Francisco, CA 94143, USA; joshua.yang@alumni.ucsf.edu (J.Y.C.Y.); arya.zarinsefat@ucsf.edu (A.Z.); reuben.sarwal@ucsf.edu (R.D.S.); tara.sigdel@ucsf.edu (T.K.S.); juliane.liberto@ucsf.edu (J.M.L.); izabella.damm@ucsfmedctr.org (I.D.); 2 KIT Bio, 2000 University Avenue, Palo Alto, CA 94303, USA; drew@kit.bio; 3 Leuven Lung Transplant Unit, Department of Chronic Diseases, Metabolism, and Ageing (CHROMETA), KU Leuven, 3000 Leuven, Belgium; stijn.verleden@kuleuven.be (S.E.V.); bart.vanaudenaerde@kuleuven.be (B.M.V.); robin.vos@uzleuven.be (R.V.); geert.verleden@kuleuven.be (G.M.V.)

**Keywords:** cfDNA, CXCL10, lung transplantation, allograft dysfunction, CLAD, BAL

## Abstract

Standard methods for detecting chronic lung allograft dysfunction (CLAD) and rejection have poor sensitivity and specificity and have conventionally required bronchoscopies and biopsies. Plasma cell-free DNA (cfDNA) has been shown to be increased in various types of allograft injury in transplant recipients and CXCL10 has been reported to be increased in the lung tissue of patients undergoing CLAD. This study used a novel cfDNA and CXCL10 assay to evaluate the noninvasive assessment of CLAD phenotype and prediction of survival from bronchoalveolar lavage (BAL) fluid. A total of 60 BAL samples (20 with bronchiolitis obliterans (BOS), 20 with restrictive allograft syndrome (RAS), and 20 with stable allografts (STA)) were collected from 60 unique lung transplant patients; cfDNA and CXCL10 were measured by the ELISA-based KIT assay. Median cfDNA was significantly higher in BOS patients (6739 genomic equivalents (GE)/mL) versus STA (2920 GE/mL) and RAS (4174 GE/mL) (*p* < 0.01 all comparisons). Likelihood ratio tests revealed a significant association of overall survival with cfDNA (*p* = 0.0083), CXCL10 (*p* = 0.0146), and the interaction of cfDNA and CXCL10 (*p* = 0.023) based on multivariate Cox proportional hazards regression. Dichotomizing patients based on the median cfDNA level controlled for the mean level of CXCL10 revealed an over two-fold longer median overall survival time in patients with low levels of cfDNA. The KIT assay could predict allograft survival with superior performance compared with traditional biomarkers. These data support the pursuit of larger prospective studies to evaluate the predictive performance of cfDNA and CXCL10 prior to lung allograft failure.

## 1. Introduction

In lung transplantation (LTx), while survival after transplantation has increased over time due to improved survival in the early post-transplant period, chronic allograft rejection remains a major cause of morbidity and mortality [1]. Infections and chronic lung allograft dysfunction (CLAD) are major reasons for this inferior long-term outcome [2]. Furthermore, the diagnosis of CLAD has proven challenging and evaluation typically includes bronchoscopy, mostly to rule out other causes such as infection. However, the sensitivity of transbronchial biopsy for the diagnosis of chronic rejection is only 30% [3]. Given the importance of diagnosing graft rejection and dysfunction, many investigators have explored the use of biomarkers in bronchoalveolar lavage (BAL) primarily in acute rejection (AR) [4,5,6,7] as an adjunct to diagnosis, but there are as yet no universally accepted diagnostic or prognostic BAL biomarkers for acute rejection nor for CLAD diagnosis or biomarkers for prediction of lung transplant survival [8].

C-X-C motif chemokine 10 (CXCL10) is increasingly being explored as a biomarker for the early detection and monitoring of renal function in renal transplant patients [9]. CXCL10 is an interferon gamma-induced, small cytokine, belonging to the C-X-C motif chemokine family. Previous studies have demonstrated that CXCL10 is a potent chemoattractant for various immune cells including CD4 and CD8. Additionally, elevated CXCL10 reflected tubulointerstitial inflammation and peritubular capillaritis [10]. Donor-derived cell-free DNA (cfDNA) has also been increasingly explored in the transplant space, especially for the detection of acute rejection in renal transplantation [11]. Measurements of donor-specific cfDNA in the plasma [12,13] and urine [14] (specifically in renal transplantation) have been shown to detect episodes of acute rejection. These methods typically require extensive sequencing, high throughput SNP-testing, or a priori knowledge of specific genetic differences between donor and recipient in order to work.

Cell-free DNA consists of fragments of nucleic acids that circulate in the biofluids of both healthy individuals and patients with a variety of diseases [15,16]. Multiple studies have shown that circulating plasma and urine cfDNA is elevated in transplant patients undergoing rejection of allografts [11,14,17]. Recent studies have utilized digital PCR to quantify donor-derived cfDNA from blood and demonstrated that donor-derived cfDNA could be used as an early non-invasive biomarker for acute lung allograft rejection [18,19]. In this study we explored the use of cfDNA and CXCL10 derived from BAL specimens as diagnostic biomarkers of CLAD and possibly as prognostic markers of overall survival.

## 2. Materials and Methods

### 2.1. Study Design

This was a retrospective analysis of BAL samples from lung transplant recipients who had transplant surgeries at the KU Leuven Division of Pneumology. The study was approved by the local hospital’s ethical committee (S58926). All patients provided written informed consent to participate in lavage biobanking and research (S51577), in full adherence to the Declaration of Helsinki.

### 2.2. Study Population and Samples

BAL specimens from 60 lung transplant recipients from the KU Leuven Division of Pneumology who were characterized as either (1) stable (*n* = 20), (2) bronchiolitis obliterans (BOS) [20] (*n* = 20), or (3) restrictive allograft syndrome (RAS) (*n* = 20) were analysed in this study. Patients were defined as stable if they lacked clinical evidence of any disease. Phenotypes of CLAD (either RAS or BOS) were diagnosed and differentiated according to histology, allograft function and imaging as previously described by our group [21]. The advantage of the lung transplant setting is that serial spirometry is performed. In most centres, it is not the routine practice to assess DLCO at every out-patient clinic. In the lung transplant setting, FEF25-75 values were used to assess airway obstruction. Additional measurements included the peak expiratory flow (PEF) values, maximum inspiratory flow (MIF) values, and DLCO.

BAL at our centre is performed routinely as part of follow-up after lung transplantation at days one, 21, 90, 180, 360, 540, and 720 post-LTx, and additionally as indicated when infection or acute/chronic rejection is suspected. As controls, BAL samples from post-operative day 720 in patients without evidence of any disease and who were CLAD-free until at least January 2017 were used. BAL samples were also available at CLAD diagnosis. BAL procedure was performed as previously described [1]. Briefly, at our centre, BAL is performed with two aliquots of 50 mL of sterile saline, of which the recovered fractions are pooled following gentle aspiration. BAL was used for differential cell count, microbiology, virology, and biobanked for future protein and cfDNA analysis.

### 2.3. Biomarker Measurement in BAL Samples

For this study, supernatants were defrosted and centrifuged at 2000 ×*g* for 30 min at 4 °C prior to assaying. For ELISA-like measurement of cfDNA in the KIT assay, a proprietary 5’ biotinylated oligonucleotide complementary chemiluminescent immunoprobe to the ALU human element was used for the measurement of specific target cfDNA fragments. Streptavidin-HRP (R&D Systems, Minneapolis, MI, USA) and SuperSignal^TM^ ELISA Femto Substrate (Thermo Fisher Scientific, Waltham, MA, USA) were used for luminescent detection and quantitation. The reported cfDNA values were dilution-adjusted and reported as genomic equivalents (GE) per mL, where one GE is equivalent to 6.6 pg of human DNA. CXCL10 was measured using a custom generated human CXCL10 ELISA. Commercial ELISA kits for IL-6 (Lifesciences) and IL-8 (Lifesciences) were used to test samples in duplicate according to manufacturer’s instructions. The absolute count and proportion of leukocytes in the BAL fluid were measured by differential cell count.

### 2.4. Statistical Analyses of cfDNA and CXCL10 with CLAD Phenotype and Overall Survival

In each sample, the listed biomarkers were measured and correlated with CLAD phenotype and overall survival. Where applicable, all statistical tests were two-sided. A *p*-value < 0.05 was considered significant. All pairwise comparisons were analysed using a one-way ANOVA followed by multiple comparisons correction by the Tukey method [22]. To assess the contributions of each biomarker, multivariate Cox proportional hazards regression was performed. Assessment of overall survival was conducted using the Cox proportional hazards regression analysis. Statistical analyses for ANOVAs and pairwise comparisons were performed in GraphPad Prism 8.0.1 (GraphPad Software, San Diego, CA, USA). Statistical analyses for receiver operating characteristic (ROC) curves and survival analyses were performed using R version 3.4.1 (R Foundation, Vienna, Austria).

## 3. Results

### 3.1. Patients and BAL Samples

For this study, a total of 60 BAL samples were collected from 60 unique lung transplant recipients. All samples were clinically annotated and consisted of 20 samples collected from patients with restrictive allograft syndrome (RAS), 20 samples with bronchiolitis obliterans (BOS), and 20 samples with normal, stable allografts (STA). A summary of demographic information and sample characteristics are provided in Table 1. 

### 3.2. Traditional Biomarkers in Lung Transplant Recipients

**Figure 1:**
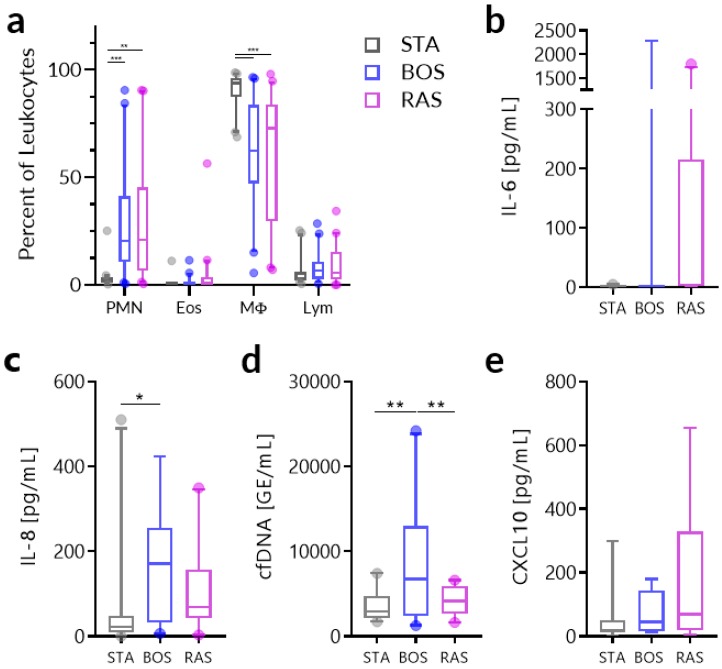
Biomarker measurements in bronchoalveolar lavage (BAL) samples among stable, restrictive allograft syndrome (RAS), and bronchiolitis obliterans (BOS) phenotypes. (**a**) Distribution of the proportion of leukocyte subsets in BAL fluid. (**b**) Distribution of IL-6 measurements, (**c**) IL-8 measurements, (**d**) cfDNA measurements, and (**e**) cfDNA measurements by chronic lung allograft dysfunction (CLAD) diagnosis. Values are shown as median with 5%–95% range. Analysis was done using two-way ANOVA for leukocyte proportions and one-way ANOVA for all other biomarkers. * *p* < 0.05, ** *p* < 0.01, *** *p* < 0.001. PMN, polymorphonuclear neutrophils; Eos, eosinophils; MΦ, macrophages; Lym, lymphocytes.

Traditional biomarkers, including IL-6, IL-8, and neutrophil proportion have been evaluated in other studies [23] and we investigated their utility in differentiating between stable and CLAD phenotypes. The distribution of the proportion of neutrophils, eosinophils, macrophages, and lymphocytes in the BAL fluid of patients by CLAD diagnosis is provided in Figure 1a. Repeated measures ANOVA revealed significant differences in the neutrophil proportion between STA and BOS (*p* = 0.0010) and STA and RAS (*p* = 0.0037) and in the macrophage proportion between STA and BOS (*p* = 0.0002) and STA and RAS (*p* = 0.0008). There were no significant differences found in the eosinophil or lymphocyte proportions. One-way ANOVA tests revealed no significant differences in the levels of IL-6 among the CLAD phenotypes (Figure 1b) while, for IL-8 (Figure 1c), only differences between STA and BOS (*p* = 0.0163) were significant. However, none of these traditional biomarkers could clearly distinguish BOS versus RAS and, in addition, substantive overlap was found for these markers across all three groups.

Subsequently, we focused our investigation toward our target biomarkers cfDNA and CXCL10. The distribution of the BAL levels of these are provided in Figure 1d,e respectively. One-way ANOVA tests revealed that cfDNA was significantly elevated between STA and BOS (*p* = 0.0025) and BOS vs. RAS (*p* = 0.0110). For CXCL10, there was a trend level difference between STA and RAS (*p* = 0.0839). 

**Figure 2:**
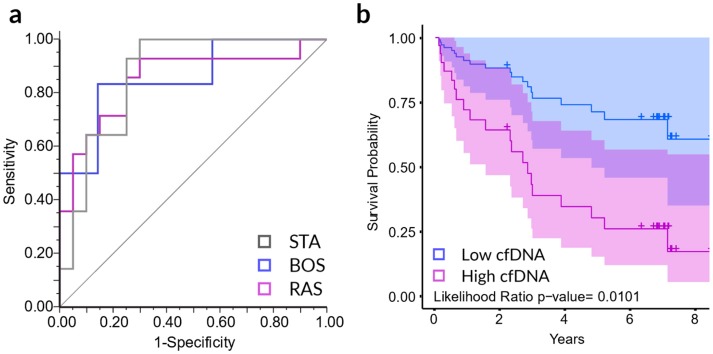
CXCL10 and cfDNA levels in BAL samples distinguish stable, RAS, and BOS phenotypes and correlate with survival probability. (**a**) The receiver operating characteristic (ROC) curve of each group versus the combination of the remaining two phenotypes as based on nominal logistic regression modeling of cfDNA, CXCL10, and the interaction of cfDNA and CXCL10. The AUCs for BOS, RAS, and stable were 0.8571, 0.8500, and 0.8679 respectively (*p* = 0.0004). (**b**) Cox proportional hazards regression analysis survival curves for low and high levels of cfDNA in BAL from CLAD patients (LR *p* = 0.0101).

Notably, cfDNA was the only biomarker measured that could distinguish between BOS and RAS CLAD phenotypes. As such, we sought to see whether the CXCL10 and cfDNA together could distinguish BOS and RAS CLAD phenotypes from each other and from stable. Nominal logistic regression of cfDNA, CXCL10, and the interaction of cfDNA and CXCL10 could distinguish BOS, RAS, and stable phenotypes from one another with a ROC AUC of 0.8571, 0.8500, and 0.8679 respectively (*p* = 0.0004) (Figure 2a). We subsequently investigated the association of these biomarkers with LTx survival using multivariate Cox proportional hazards regression to assess the degree of association of cfDNA, CXCL10, and the interaction of cfDNA and CXCL10 with overall survival in LTx patients. Likelihood ratio tests revealed a significant association of overall survival and cfDNA (*p* = 0.0083), CXCL10 (*p* = 0.0146) and the interaction of cfDNA and CXCL10 (*p* = 0.023). We further evaluated the utility of the traditional biomarkers IL-6, IL-8, and leukocyte proportions in predicting survival in the context of cfDNA and CXCL10. Based on likelihood ratio statistics for hierarchical proportional hazards models, neither IL-8 (LR *p* = 0.3556), nor arcsine transformed percent neutrophils (LR *p* = 0.0736), provided significant information above and beyond cfDNA, CXCL10 and the interaction of cfDNA and CXCL10 alone. In addition, there was no significance between CLAD diagnosis for percent macrophages (LR *p* = 0.1140), percent lymphocytes (LR *p* = 0.3568), or percent eosinophils (LR *p* = 0.5812) in BAL. While IL-6 alone provided some prediction for overall survival (LR *p* = 0.0235), cfDNA, CXCL10 and the interaction of cfDNA and CXCL10 provided significant survival prediction information beyond IL-6 alone (LR *p* < 0.0001). These results suggest that cfDNA and CXCL10 may be synergistic predictors of CLAD patient survival.

Figure 2b provides the Cox proportional hazards regression survival plot dichotomizing LTx patients by median cfDNA level into two groups of low and high cfDNA patients controlling for the mean level of CXCL10. These results show that the median overall survival time increases over two-fold in patients with low levels of cfDNA at CLAD diagnosis (*p* = 0.0101).

## 4. Discussion

In this study, we used BAL fluid samples from lung transplant patients to evaluate the predictive capacity of the biomarkers CXCL10 and cell-free DNA to detect and distinguish between subphenotypes of chronic lung allograft disorder as well as predict transplant survival. Our results showed that both CXCL10 and cfDNA together could distinguish between stable patients and the BOS and RAS subphenotypes of CLAD. Furthermore, both CXCL10 and cfDNA together could segregate patients into low- and high-survival patient populations, which superseded the utility of traditional biomarkers such as IL-6 and IL-8 in their predictive capacity.

Such findings are important because, of all major solid organ transplants, lung transplantation has the worst median overall survival of approximately 6 years as compared to >10 years for both heart and kidney transplantation [24,25,26]. This is largely due to the development of CLAD in 50% of recipients after 5 years, ultimately leading to significant morbidity and mortality. The mechanisms underlying CLAD development are poorly understood in part because a diagnosis of CLAD describes not a single entity, but a heterogeneous group of phenotypes characterized by varying degrees of airway neutrophilia, fibrosis, histological features, and responsiveness to therapy [27]. While two major phenotypes of CLAD have been described, BOS and RAS [21], the differential phenotyping of these two is confounded by a lack of biomarkers able to effectively differentiate the two [8]. Identification of such biomarkers is essential because they may elucidate mechanistic differences between these phenotypes and lead to the development of individual therapies specific to each condition.

Our group and others have sought to identify such biomarkers in the BAL fluid of LTx patients, as collection of this biofluid is relatively noninvasive and directly reflects the pulmonary milieu. Biomarkers in the BAL fluid such as neutrophil proportions [23,27], IL-6 [28,29], and IL-8 [23,28], have been found to differentiate between CLAD and stable phenotypes, but do not reliably distinguish between BOS and RAS. As such, additional biomarkers are necessary to understand the differences 

The current study is the first study to jointly assess CXCL10 and cfDNA in BAL. CXCL10, a pro-inflammatory cytokine that is a CXCR3 ligand, has been previously found to be associated with diffuse alveolar damage in and development of CLAD [30] and also as a risk factor for CLAD development in the context of organizing pneumonia post-transplantation [31]. Previously, our group found that CXCL10 was elevated in RAS versus stable patients and was associated with survival [28]. While donor-derived cfDNA has been evaluated in the peripheral blood of LTx patients for monitoring infection, acute rejection, and CLAD [17], levels of cfDNA in the BAL fluid of LTx patients has not been explored.

In the BAL of LTX patients, the combination of cfDNA and CXCL10 were found not only to differentiate between BOS and RAS patients, but also associate with graft survival after diagnosis. This suggests that high levels of both may identify patients with a poor prognosis. Our data show that BOS patients had a significantly greater amount of cfDNA in BAL than stable or RAS patients. Additionally, BOS patients had greater neutrophil and IL-8 counts compared to stable patients. Given the role of IL-8 as a neutrophil chemotactic factor, it is not surprising to see its levels elevated in the BOS group, who also had elevated neutrophils counts. The elevated presence of cfDNA in the BAL of BOS patients is likely multifactorial and we hypothesize it is likely from a combination of tissue injury in CLAD and release from granulocytic inflammatory cells, such as that in the form of neutrophil extracellular traps, resulting in both donor-derived and host cfDNA.

Given that CXCL10 was not significant in univariate analysis, its key role in differentiating between the different phenotypes and in predicting survival in the context of cfDNA was surprising. DNA, through activation of the TLR9 receptor, has been shown to be upstream of CXCL10 release by BAL fluid cells [32]. Given that CXCL10 can bind to CXCR3 on neutrophils and this receptor-ligand interaction has been shown to enhance neutrophil activation and neutrophil-mediated lung injury [33], it is possible that cfDNA and CXCL10 work cyclically to amplify inflammation and contribute to CLAD development. Mechanistic studies are needed to elucidate the exact roles that each of these biomarkers play in the post-transplantation pulmonary environment. Such studies could elucidate the relationship between these biomarkers and the differences in survival and help to develop targeted therapeutics to prevent CLAD development or progression.

Limitations of this study include the small sample size but, as an exploratory study, our results suggest cfDNA and CXCL10 measurements in BAL as highly synergistic biomarkers could be utilized prognostically in in LTx patients. These markers together appear to provide useful information above and beyond standard BAL biomarkers including IL-6, IL-8, and neutrophils. Larger prospective studies in LTx patients utilizing cfDNA and CXCL10 are needed to further assess our findings here and are currently underway. Future studies with more robust characterization of serial cfDNA and CXCL10 levels in BAL samples from lung transplant could lead to a biomarker to help clinicians anticipate the development of CLAD in lung transplant recipients, and/or monitor its progression and the relative effectiveness of current and future therapeutic interventions.

## Figures and Tables

**Table 1:** Demographics and characteristics ^a^.

Phenotype Characteristic	Stable (20 samples)	BOS (20 samples)	RAS (20 samples)
Recipient			
• Recipient age, year (SD)	46 (14)	51 (15)	44 (16)
• Recipient male/female, %	35	40	50
Donor			
• Donor age, year (SD)	39 (15)	47 (14)	46 (13)
• Donor male/female, %	40	45	50
Indication for lung transplantation, %			
• Emphysema	35	35	5
• COPD	0	15	40
• Cystic fibrosis	30	15	25
• Pulmonary Fibrosis	20	5	10
• PHT ^b^	0	0	10
• PPH ^b^	5	5	0
• Other ^c^	10	25	10
Spirometry			
• TLCO	5.25 (1.49)	5.08 (2.18)	3.61 (1.56)
• PEF	7.75 (2.15)	5.51 (2.06)	5.01 (1.74)
• FEF (25–75)	2.79 (1.36)	1.04 (0.83)	1.17 (0.92)
• MIF	4.55 (1.90)	3.91 (1.08)	3.60 (1.26)
• FEV1/FVC ratio, %	82 (9)	58 (13)	69 (17)
Overall survival, days	2569 (215)	1009 (526)	434 (376)

^a^ Values are reported in the given units with standard deviation in parentheses. Characteristics and demographic information of recipients is based on the day of bronchoalveolar lavage (BAL) collection. Demographic information of donors is based on the day of lung transplant. ^b^ One bronchiolitis obliterans (BOS) patient with PPH was diagnosed with PAH. Two restrictive allograft syndrome (RAS) patients had a PHT diagnosis, one with PAH and one with congenital heart syndrome. One stable patient with PPH was diagnosed with Eisenmenger syndrome. ^c^ Other indications included: William Campbell, histiocytosis X, NSIP, UIP, sarcoidosis, asthma, Alpha-1, LAM, and BRECT. BOS, bronchiolitis obliterans. RAS, restrictive allograft syndrome. COPD, chronic obstructive pulmonary disease; PPH, primary pulmonary hypertension; Alpha-1, alpha-1 antitrypsin deficiency; NSIP, nonspecific interstitial pneumonia; UIP, usual interstitial pneumonia; PHT, pulmonary hypertension; LAM, lymphangioleiomyomatosis. BRECT, bronchiectasis; TLCO, transfer factor; PEF, peak expiratory flow; FEF (25–75), forced expiratory flow at 25%–75%; MIF, maximum inspiratory flow.
